# Genetic admixture and lineage separation in a southern Andean plant

**DOI:** 10.1093/aobpla/plw034

**Published:** 2016-07-11

**Authors:** Santiago Morello, Silvana M. Sede

**Affiliations:** Instituto de Botánica Darwinion IBODA-ANCEFN-CONICET, Labardén 200, San Isidro, Buenos Aires, B1642HYD, Argentina

**Keywords:** AFLP, *Escallonia*, evolution, genetic diversity, leaf shape, Patagonia, plastid DNA sequences, Southern Andes

## Abstract

Mountain uplifts have generated new ecologic opportunities for plants, and triggered evolutionary processes, favouring an increase on the speciation rate in all continents. Moreover, mountain ranges may act as corridors or barriers for plant lineages and populations. In South America a high rate of diversification has been linked to Andean orogeny during Pliocene/Miocene. More recently, Pleistocene glacial cycles have also shaped species distribution and demography. The endemic genus *Escallonia* is known to have diversified in the Andes. Species with similar morphology obscure species delimitation and plants with intermediate characters occur naturally. The aim of this study is to characterize genetic variation and structure of two widespread species of *Escallonia*: *E. alpina* and *E. rubra*. We analyzed the genetic variation of populations of the entire distribution range of the species and we also included those with intermediate morphological characters; a total of 94 accessions from 14 populations were used for the Amplified Fragment Length Polymorphism (AFLP) analysis. Plastid DNA sequences (*trnS-trnG*, *3*′*trnV-ndhC* intergenic spacers and the *ndhF* gene) from sixteen accessions of *Escallonia* species were used to construct a Statistical Parsimony network. Additionally, we performed a geometric morphometrics analysis on 88 leaves from 35 individuals of the two *E. alpina* varieties to further study their differences. Wright’s Fst and analysis of molecular variance tests performed on AFLP data showed a significant level of genetic structure at the species and population levels. Intermediate morphology populations showed a mixed genetic contribution from *E. alpina* var. *alpina* and *E. rubra* both in the Principal Coordinates Analysis (PCoA) and STRUCTURE. On the other hand, *E. rubra* and the two varieties of *E. alpina* are well differentiated and assigned to different genetic clusters. Moreover, the Statistical Parsimony network showed a high degree of divergence between the varieties of *E. alpina*: var. *alpina* is more closely related to *E. rubra* and other species than to its own counterpart *E. alpina* var. *carmelitana*. Geometric morphometrics analysis (Elliptic Fourier descriptors) revealed significant differences in leaf shape between varieties. We found that diversity in *Escallonia* species analyzed here is geographically structured and deep divergence between varieties of *E. alpina* could be associated to ancient evolutionary events like orogeny. Admixture in southern populations could be the result of hybridization at the margins of the parental species’ distribution range.

## Abstract (precis)

Mountain orogeny has been a major factor in plant evolution in all continents by changing the landscape and climate, creating new habitats and ecological opportunities. In this study we found that diversity in two southern Andean *Escallonia* species is geographically structured and there is a deep divergence between infraspecific groups that could be associated with ancient evolutionary events like orogeny. We also found evidence of admixture, likely the result of hybridization at the margins of the parental species' distribution range.

## Introduction

Mountain orogeny has been a major factor in plant evolution in all continents, and has been linked to recent diversification and speciation events (Hughes and Atchison 2015). The uplift of mountain ranges may change the landscape and climate, creating different environments and microclimates that provides new habitats (Linder 2008) and island-like ecological opportunities (Hughes and Eastwood 2006).

In South America, the Andes mountains have played these roles since their uplift during Pliocene/Miocene (16–5.3 Mya). Moreover, the Andes themselves could have played as a North-South corridor allowing the exchange of plant lineages; or as a barrier promoting vicariance. Consequently, these evolutionary processes triggered by the Andean uplift promoted a high speciation rate conducting to great biological diversity in South America (Antonelli *et al.* 2009; Hartley 2003; Kier *et al.* 2009; Luebert *et al.* 2011; Ortiz-Jaureguizar and Cladera 2006; Scherson *et al.* 2008).

The southern Andes have been identified as the origin of diversification of many groups (e.g. *Chuquiraga*: Ezcurra 2002, *Valeriana*: Bell *et al.* 2012, *Oxalis*: Heibl and Renner 2012, *Heliotropium*: Luebert *et al.* 2011); they are located in Argentina and Chile from 29° S, below Atacama desert to the austral tip of the continent at the Magellan and Fuegian Archipelagos.

During Pleistocene, climate oscillations have greatly influenced biodiversity in all continents (Hewitt 2000, 2004). In Patagonia, glacial cycles generated not only warmth-cold fluctuations in climate but also ice sheet expansions and retreats modelling landscape (Rabassa *et al.* 2011). As a consequence, plant species have accompanied those changes reacting with population contractions and expansions, long-distance range dispersal, new habitat colonization and *in situ* survival in refugia; examples of these processes have been studied for many Andean plants (e.g. *Austrocedrus chilensis*: Pastorino *et al.* 2004; Souto *et al.* 2012, 2015; *Nothofagus* species: Marchelli and Gallo 2006; Premoli *et al.* 2010; Soliani *et al.* 2012; *Hordeum* species: Jakob *et al.* 2009; *Hypochaeris incana*: Tremetsberger *et al.* 2009; *Calceolaria polyrhiza*: Cosacov *et al.* 2010, *Eucryphia cordifolia*; Segovia *et al.* 2012). Common processes for different lineages are suggested by two shared patterns a) high diversity zones corresponding to putative glacial refugia; and b) latitudinal phylogeographical breaks along the Andes and Patagonian steppe (Sérsic *et al.* 2011).

Among the plant lineages from South American mountain regions, the endemic genus *Escallonia* (Sede 2008; Sleumer 1968) is distributed along the whole Andean mountain range where it is highly diverse (Sleumer 1968; Sleumer and Correa 1984), and in different ecosystems in southern Brazil and central Argentina. *Escallonia* is the most numerous genus of the family Escalloniaceae comprising *ca*. 40 species; plant morphology displays high variation among species, and many diagnostic characters also show intraspecific variability (e.g. size and shape of leaves and floral organs, petal pigmentation, and presence of hairs and glands in different organs). Moreover, plants with intermediate morphology (IM) between species have been described (Sleumer 1968). Current taxonomy matches this pattern, with some species descriptions containing overlapping characters and *ca.* 20 species varieties described (Kausel 1953; Sleumer 1968; Sleumer and Correa 1984).

Molecular phylogenetic studies in *Escallonia* corroborated the monophyly of the genus. Five lineages were strongly supported and geographically structured, suggesting that their evolutionary history might be linked to orogenic processes in South America (Sede *et al.* 2013; Zapata 2013). Particularly in the southern Andes, two independent lineages were evident (Sede *et al.* 2013): clade B with only two species (*Escallonia* *virgata*, restricted to southern Andes and *Escallonia* *callcottiae*, endemic of Juan Fernandez archipelago in Chile) and clade D with the remaining nine species sampled (*E.* *alpina*, *E.* *florida*, *E.* *illinita*, *E.* *leucantha*, *E.* *myrtoidea*, *E.* *pulverulenta*, *E.* *revoluta*, *E.* *rosea* and *E.* *rubra*). Within this major lineage, two species, *E.* *alpina* and *E. rubra*, are highly polymorphic and share the same distribution range along the Andes in Patagonia, although they only differ in maximum elevation: *E. alpina* reaches higher altitudes (up to ca. 2200 m) than *E. rubra*, which occurs up to ca. 1700 m. These species are differentiated mainly by the distribution of glands in leaves and flowers and by the type of inflorescence. For both species some varieties have been described on the basis of morphological characters and geographical distribution. There are two varieties described for *E. alpina* based on vegetative characters: *E. alpina* var. *carmelitana* only differs from the type variety in the colour of the stem and leaf size. A preliminary study on the genetic variability, including five populations, supported species boundaries and one population showing intermediate morphological characters was detected and corroborated by AFLP patterns (Morello *et al.* 2013).

The occurrence of individuals with IM between *E. alpina* and *E. rubra* [described as hybrids by Kausel (1953); Sleumer (1968) and Sleumer and Correa (1984)] along with the description of a population with genetic admixture (Morello *et al.* 2013) and the lack of species exclusivity in phylogenetic reconstructions (Zapata 2013), indicate the presence of *Escallonia* hybrids in Patagonia.

Hybridization is a common phenomenon in plants and there are different views on its role in evolution: from not significant (Mayr 1992) to very relevant in speciation (Rieseberg 1997; Mallet 2007) or in adaptation to new environments (Rieseberg and Wendel 1993). Intermediate morphology is a strong indication of mixed ancestry (Stebbins 1959; Stace 1991); although hybrids are not always morphological intermediates (Rieseberg and Ellstrand 1993) and morphological intermediate individuals are not always hybrids (Morrell and Rieseberg 1998; Park *et al.* 2003). Hybridization has been frequently documented in the southern Andes (e.g. *Fuchsia*: Berry 1982; *Discaria*: Tortosa 1983) but there are few works that explore its evolutionary relevance in this region (*Calceolaria*: Sérsic *et al.* 2001; *Caiophora*: Ackermann *et al.* 2008; *Nothofagus*: Acosta and Premoli 2010; Soliani *et al.* 2012). A better understanding of natural hybrids between *Escallonia* species will elucidate taxonomic problems in the genus and will provide new hypotheses on its evolution.

Our aims are to characterize the genetic variation between *E. rubra* and *E. alpina*, by means of plastid DNA sequences and Amplified Fragment Length Polymorphism (AFLP). Additionally two southernmost populations with intermediate morphological characters between both species are included to investigate the genetic bases of their variation. We further analyze morphological variation in *E. alpina* (including the broadly distributed var. *alpina* and var. *carmelitana*, restricted to north Patagonia) using a geometric morphometrics analysis of leaf shape. We predict that *E. rubra*, *E. alpina* var. *alpina* and *E. alpina* var. *carmelitana* will have a high degree of genetic differentiation, while populations with intermediate morphology will show genetic admixture. Finally, leaf shape will be useful to differentiate the two varieties of *E. alpina*.

## Methods

### Plant material

**Figure 1.**
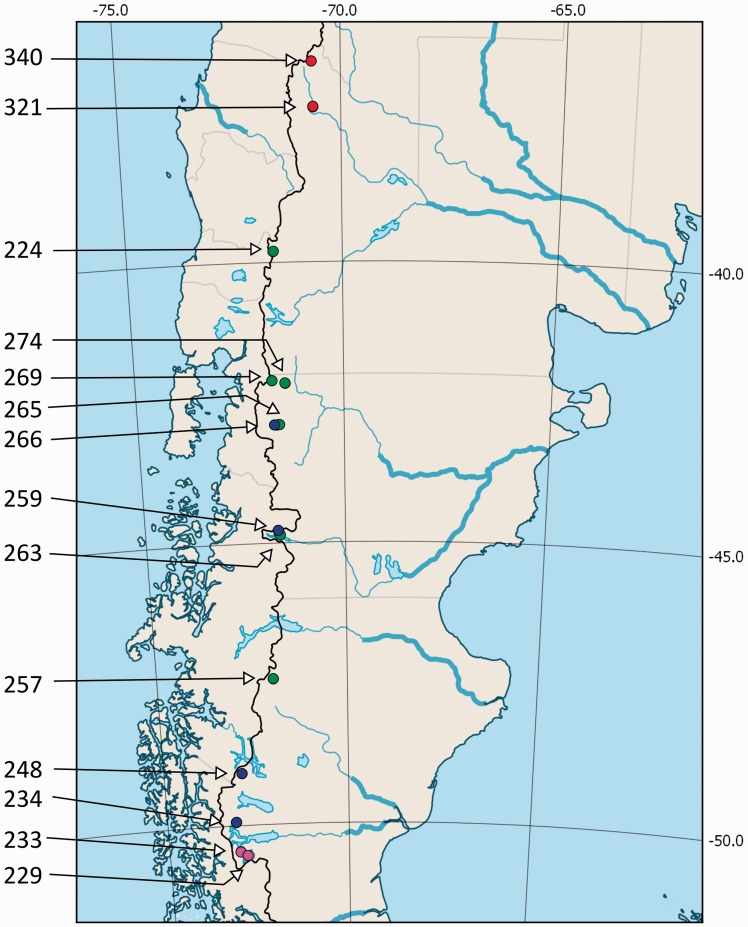
Geographical location of the 14 populations of *Escallonia* sampled, as listed in Table 1. Colours indicate groups according to taxonomy and morphological identifications: red: *E. alpina* var. *carmelitana*; green: *E. rubra*; blue: *E. alpina* var. *alpina*; fuchsia: IM.

**Figure 2.**
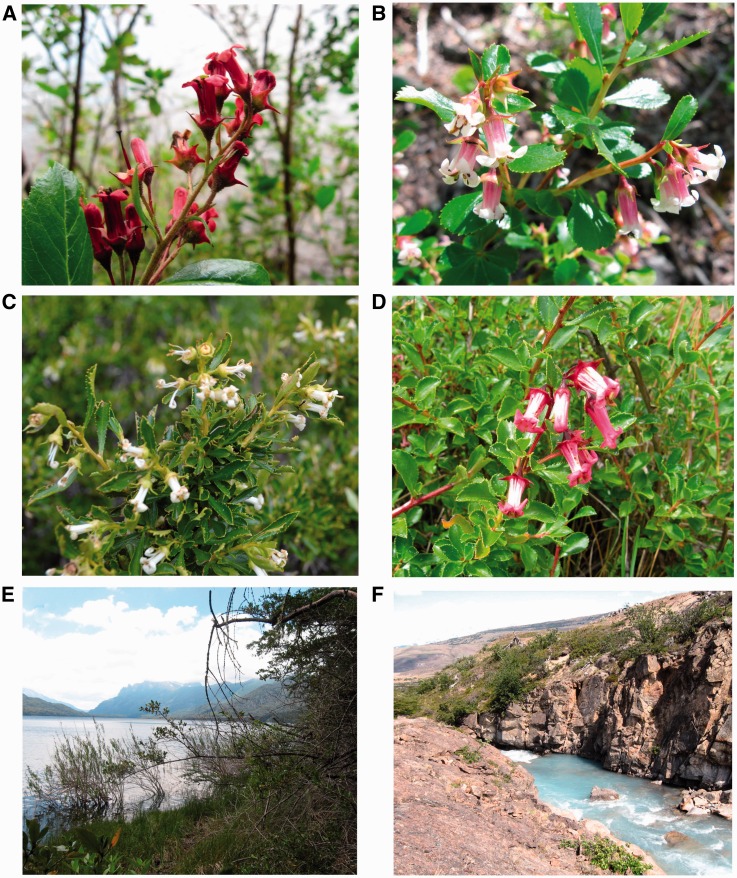
Inflorescences. (A) *E. rubra* (265). (B) *E. alpina* var. *alpina* (266). (C) *E. alpina* var. *carmelitana* (340). (D). IM population (229). Typical habitat. (E) *E. rubra* (224), Lago Carilaufquen. (F) *E. alpina* (234), Chorrillo Los Perros, Estancia Cristina.

Collection trips were undertaken on the eastern side of the Andes, in the Argentinean patagonian region (Table 1; Figs. 1 and 2). Six populations of *E.* *rubra* were collected from 39° to 47° south latitude (S) in Neuquén, Chubut and Santa Cruz provinces, four of *E.* *alpina* var. *alpina* from 42° to 49° S, in Chubut and Santa Cruz provinces, and two of *E. alpina* var. *carmelitana* in northern Neuquén province, from 36° to 37° S. Two additional populations with intermediate morphological characters were collected at *ca.* 50° S in southwestern Santa Cruz province (Table 1; Figs. 1 and 2). Herbarium material was deposited at SI (Thiers 2015).

**Table 1.** *Escallonia* populations included in this study: voucher, geographical location, coordinates, and altitude. Abbreviations: *n*, number of individuals analyzed; PNLG, Parque Nacional Los Glaciares; PNLA, Parque Nacional Los Alerces; PNL, Parque Nacional Lanín; PNLP, Parque Nacional Lago Puelo.

Species	Voucher number	*n*	Geographical location	Geographical coordinates	Altitude
*E. alpina* var. *alpina*					
	SS 234	5	Santa Cruz. PNLG, Secc. Guanaco, Estancia Cristina	49° 56′ 37′′ S 73° 06′ 46′′W	299 m
	SS 248	7	Santa Cruz. PNLG, Secc. El Chaltén, Cerro Polo	49° 17′ 57′′ S 72° 54′ 11′′ W	700 m
	SS 259	6	Chubut. Lago Fontana, Cascada de La Virgen	44° 49′ 18′′ S 71° 39′ 33′′ W	1020 m
	SS 266	8	Chubut. PNLA, Cerro Dedal	42° 54′ 01′′ S 71° 38′ 19′′ W	1126 m
*E. alpina* var. *carmelitana*					
	SS 321	4	Neuquén. Cordillera del Viento, Arroyo Manzanito	37° 14′ 38′′ S 70° 37′ 09′′ W	1455 m
	SS 340	5	Neuquén. Naciente del Río Neuquén	36° 25′ 18′′ S 70° 38′ 40′′ W	1834 m
*E. rubra*					
	SS 224	5	Neuquén. PNL, Cascada Carilaufquen	39° 48′ 29′′ S 71° 36′ 09′′ W	888 m
	SS 263	8	Chubut. 25 km al sur de Corcovado	44° 50′ 20′′ S 71° 38′ 54′′ W	688 m
	SS 265	8	Chubut. PNLA, Lago Menéndez, Puerto Chucao	42° 53′ 40′′ S 71° 35′ 13′′ W	541 m
	SS 269	7	Chubut. 31 km al S de El Bolsón	42° 09′ 43′′ S 71° 24′ 07′′ W	273 m
	SS 274	8	Chubut. PNLP. Arroyo Los Hitos	42° 06′ 19′′ S 71° 43′ 23′′ W	201 m
	SS 257	8	Santa Cruz. Camino a puesto de Gendarmería, Río Oro	47° 25′ 07′′ S 71° 56′ 36′′ W	273 m
IM					
	SS 229	8	Santa Cruz. PNLG, Secc. Lago Roca, Cerro de los Cristales	50° 32′ 32′′ S 72° 47′ 55′′ W	396 m
	SS 233	7	Santa Cruz. PNLG, Secc. Glaciar P. Moreno, Lago Argentino	50° 27′ 40′′ S 73° 01′ 36′′ W	178 m

For population genetic analyses we collected eight individuals from each location. Individuals were collected at least 15 m apart in order to avoid collecting close relatives. When eight individuals were not available, at least four were collected for each population. Fresh leaves were separated and dried with silica gel.

For morphological analyses we used pressed leaves of *E. alpina* including individuals from both varieties used in the population genetic analyses and additional herbarium material identified either as var. *alpina* or var. *carmelitana* **[See Supporting Information 1a]**.

### Total DNA isolation

Genomic DNA was extracted from silica dried leaves following a cetyltrimethylammonium bromide (CTAB) protocol (Doyle and Doyle 1987) with some modifications. Twenty µg of dried leaves were cooled by immersing in liquid nitrogen and then ground into fine powder. The samples were transferred to 1.5mL tubes with 700 µL warm CTAB buffer [2 % CTAB, 100 mM Tris–HCl pH 8.0, 1.4 M NaCl, 20 mM EDTA, 2 % polyvinylpyrrolidone] and kept in a water bath at 65 °C for an hour with continuous shaking and mixing by inversion. Tubes were then removed from the water bath and left to cool at room temperature for 10 min, then 700 µL of chloroform: isoamyl alcohol (24:1) was added and the contents were mixed by vortexing. Centrifugation was carried out at 12 000 rpm for 1 min and the aqueous (top) layer transferred into a new 1.5 mL tube. A solution of NaCl (5M; 300 µL) and ice-cold isopropanol (600 µL) were added and mixed gently by inversion; tubes were incubated for 30 min at −20 °C. Centrifugation was carried out at 12 000 rpm for 1 min, then the supernatant was discarded and the pellet was washed twice with ice-cold ethanol (70 %; 700 μL). After drying at room temperature, the pellet was suspended in 50 μL of 10:1 TE (10 mM Tris: 1 mM EDTA) buffer.

### Plastid DNA

#### Amplification and sequencing

The plastid intergenic spacers *trnS-trnG*, *3′trnV-ndhC* and the *ndhF* gene **[see Supporting Information 2]** were amplified with a profile consisting of 94 °C for 3 min, followed by 30 cycles of 94 °C for 1 min, 52 °C for 1 min and 72 °C for 1 min. Polymerase chain reactions (PCRs) were performed in a final volume of 25 μL with 50 ng of DNA template, 0.2 μM of each primer, 25 μM deoxynucleotide triphosphates, 5 mM MgCl2, 1× Taq buffer and 1.5 units of Taq polymerase provided by Invitrogen-Life Technologies (Brazil). Automated sequencing was performed by Macrogen Inc. (South Korea). We edited and assembled electropherograms in BioEdit 7.0.9 (Hall 1999). All new sequences were deposited in GenBank with accession numbers KU759574- KU759579. For sequence alignment, we used MAFFT v.7 (Katoh and Standley 2013) available online (http://mafft.cbrc.jp/alignment/server/ Last accessed 31 May 2016), with default settings.

#### Haplotype network

To understand the relation between the species of *Escallonia* distributed in the southern Andes, an haplotype network was constructed using statistical parsimony (0.95 probability connection limit) and the genealogical reconstruction algorithms of Templeton *et al.* (1992) as implemented in the R package pegas 0.8–1 (Paradis 2010). For this study we used new DNA sequences and sequences included in Sede *et al.* (2013) for a total of 16 individuals comprising southern Andean species: *E. alpina, E. callcottiae*, *E. florida*, *E. illinita*, *E. leucantha*, *E. myrtoidea*, *E. pulverulenta*, *E. revoluta*, *E. rosea*, *E. rubra* and *E. virgata.* We included one individual for species except in the case of *E. alpina* (5 accessions from two varieties: *E. alpina* var. *alpina* and *E. alpina* var. *carmelitana*) and *E. leucantha* (2 accessions). All three plastid regions were concatenated; in a few cases, when it was not possible to obtain sequences from the same individual, data from different individuals was combined to represent species.

## AFLP

AFLP was used to analyze the genetic variability, both at the species and population level. A total of 14 populations (94 individuals) were kept based on the presence of good-quality DNA samples: four of *E.* *alpina var.* *alpina*, two of *E. alpina var.* *carmelitana*, six of *E. rubra*, and two populations with intermediate morphological diagnostic characters between both species (IM) (Table 1, Fig. 1). AFLP protocol was performed essentially as described by Vos *et al.* (1995), with fluorescent labelled primers that allowed automatic detection of the amplified fragments. Genomic DNA samples (50–100 ng) were digested to completion with EcoRI and MseI and the fragment ends were ligated to EcoRI- and MseI-specific adaptors **[Supporting Information 2]** in a single reaction for three hours at 37 °C. The digestion-ligation products were diluted 20-fold into 10 mM Tris-HCl, 0.1 mM EDTA (pH 8.0) and amplified using EcoRI-A and MseI-C as pre-selective primers. The resulting template was diluted 20-fold prior to amplification with selective primers: EcoRI (FAM)-ACT and MseI-CAC **[Supporting Information 2]**. The fluorescence-labelled selected amplification products were separated on a sequencer with an internal size standard at Macrogen Inc. (South Korea). Nine random samples (9.6 % of individuals) were duplicated in order to assess reproducibility.

Fragment-size fluorescent data was visualized using Peak Scanner (Applied Biosystems, available at http://products.invitrogen.com/ivgn/product/4381867, last accessed 31 May 2016) and automatically scored using the R package RawGeno v2.0-1 (Arrigo *et al.* 2012 available at http://sourceforge.net/projects/rawgeno, last accessed 31 May 2016). Peaks between 70 and 500 bp with fluorescence higher than 120 relative fluorescent units (RFU) were retained and non reproducible fragments were removed. Presence or absence of each fragment was recorded for each individual obtaining a binary matrix [**Supporting Information 3**]. Pearson correlation coefficients between each fragment size and its frequency were calculated in order to assess the possibility of homoplasy (Vekemans *et al.* 2002).

### Genetic structure

The AFLP matrix was used to infer genetic structure from *Escallonia* populations in order to assess the identity of IM populations and also to investigate the relation between *E.* *alpina* varieties. Differentiation due to genetic structure was tested using Wright’s fixation index (Fst) for all populations and for separate species. Additionally a matrix of pairwise Fst genetic distances between all populations was constructed.

Principal Coordinate Analysis (PCoA, Gower 1966) was performed using Jaccard genetic distances between individuals calculated from the binary matrix using software FAMD v1.108 (Schlüter and Harris 2006); this multivariate analysis projects pairwise genetic distances upon a set of derived orthogonal axes, this reduced dimensional space allows the recognition of genetically similar individuals. Additionally, a bayesian analysis of population structure was performed using software STRUCTURE v2.3 (Falush *et al.* 2003, 2007; Pritchard *et al.* 2000) to infer the distribution and number of genetic clusters (K). The default model was used which assumes correlated frequencies between clusters and allows individuals to have a mixed ancestry. The log-likelihood probability of the data was calculated for each possible K value from 1 to 15 using 20 runs of 1 000 000 MCMC iterations following a burn in period of 100 000 iterations. Convergence of parameters between different runs was analyzed using Tracer v1.4 (Rambaut *et al.* 2013). The best fit number of clusters was calculated according to Evanno *et al.* (2005) using STRUCTURE HARVESTER (Earl and vonHoldt 2011).

We performed an analysis of molecular variance (AMOVA) using Arlequin v.3.5.1.3 (Excoffier and Lischer 2010). This analysis allowed us to assess the distribution of variance among taxonomic units (*E. rubra*, *E. alpina* var. *alpina* and *E. alpina* var. *carmelitana);* and among and within populations, and to test for significant differences.

### Leaf shape analysis of *E.* *alpina* varieties

In order to characterize both varieties of *E. alpina*, leaf shape of var. *alpina* and var. *carmelitana* was described by traditional methods, using specimens from our collections (Table 1) and herbarium material **[Supporting Information 1b].**

Additionally, leaf shape was studied by performing an Elliptic Fourier Analysis (Kuhl and Giardina 1982). A total of 88 leaves from 35 collections of *E. alpina* including var. *alpina* and var. *carmelitana* (see Table 1 and **Supporting Information 1b**) were cut, hydrated, pressed and photographed using a Cannon PowerShot G11 digital camera over a clear background. The digital files were manipulated to obtain a binary image using Fiji (Schindelin *et al.* 2012). Final digitized images are available as **Supporting Information 4**. The R package Momocs V0.9 (Bonhomme *et al.* 2014) was used to extract the outlines as coordinates and to perform succeeding analysis. Each leaf shape was reconstructed using the first 15 harmonics, this number was selected by comparing the original outline and several reconstructed shapes using an increasing harmonics number.

A principal component analysis (PCA, Hotelling 1933) was performed on the Fourier coefficients of the leaves shape, and ordination of samples was then plotted on the first two principal components (PCs) axes.

The PCs explaining >99 % of variance were used as shape variables in subsequent analyses. A Linear Discriminant Analysis (LDA, equivalent to Canonical Variate Analysis) was carried to show maximum separation among *E. alpina* varieties. The LDA matrix was tested using a leave-one-out cross validation procedure to compute the percentage of correctly assigned individuals for each variety (Martens and Dardenne 1998). Additionally, PCs were subjected to multi-variate analyses of variance (MANOVA) to evaluate the significance of the separation between the two groups.

## Results

### Sequence variation and haplotype network

**Figure 3.**
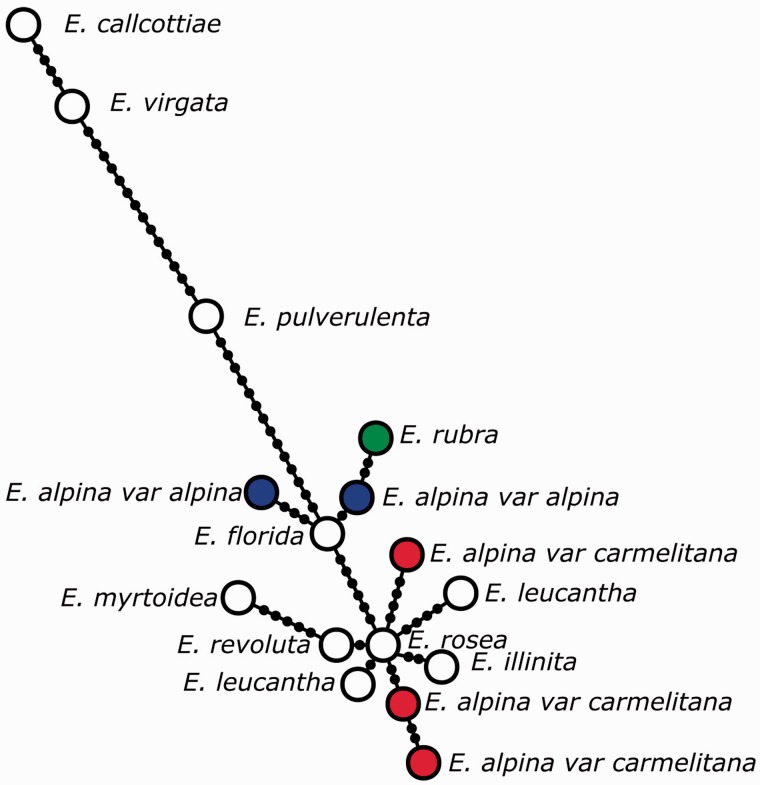
Statistical Parsimony network connecting haplotypes from southern Andean *Escallonia* species. For each haplotype we concatenated the sequences of *trnS-trnG* (740 bp), *3′trnV-ndhC* (602 bp) intergenic spacers and the *ndhF* gene (1803 bp). Each circle correspond to a unique haplotype. Dots on lines represent mutational steps.

The PCR amplification of intergenic spacers resulted in fragments of 740 bp (*trnS-trnG*) and 602 bp (*trnV-ndhC*), and the amplification of *ndhF* gene (incomplete sequence) resulted in a 1803 bp-fragment. All accessions, including multiple accessions for one species, showed exclusive haplotypes (Fig. 3).

In the network (Fig. 3), one major group is composed of *E. leucantha*, *E. illinita*, *E. rosea*, *E. revoluta*, *E. myrtoidea*, *E. alpina* (including var. *carmelitana*), *E. rubra*, and *E. florida*, which are separated by few mutational steps. The remaining species are separated by a long chain of mutational steps from the main group.

In particular, new sequences of *E. alpina* var. *alpina* and var. *carmelitana* were included in the major group together with those downloaded from the GenBank database. Varieties were grouped together, although neither of them shares a unique haplotype. Moreover, all accessions of *E. alpina* var. *alpina* are more closely related to other species like *E. rubra* and *E. florida* than they are to *E. alpina var.* *carmelitana* accessions; both varieties were separated by at least 7 mutational steps.

### AFLP

Non replicated peaks (4.8 %) were eliminated and a matrix with 174 fragments for the 94 individuals sampled was obtained. Linear regression of fragment size and fragment frequency was not significant (*R*^2^ = −0.153; *P* = 0.07). *Escallonia rubra* and *E. alpina* (including both varieties) had, respectively, 7 and 19 exclusive fragments. At the same time *E. alpina* var. *alpina* and *E. alpina* var. *carmelitana* had seven exclusive fragments each.

### Genetic structure

When comparing all populations, Wright’s fixation index showed a significant degree of genetic structure (Fst = 0.17, *P* < 0.001). As this Fst value encompassed differences among taxonomic units we performed separate analyses for *E. rubra* (Fst = 0.13, *P* < 0.001) and *E. alpina* var. *alpina* (Fst = 0.03, *P* = 0.002). *Escallonia* *rubra* showed a much higher level of genetic structure, although both were significantly different from zero.

Pairwise differences among populations are shown in Table 2. Fst distances were generally low when comparing populations from the same taxonomic unit (Fst = 0.01–0.11) and higher when comparing populations from different taxonomic units (Fst = 0.10–0.39). The notable exception was population 224 that showed high Fst values (0.2–0.39) when compared with any other population. The two populations with IM showed low levels of genetic differentiation when compared to populations of *E. alpina* var. *alpina* and *E. rubra* (Fst = 0.01–0.13) but higher values when compared to *E. alpina* var. *carmelitana* (0.17–0.22).

**Table 2:** Pairwise genetic differentiation (Fst) between populations of *Escallonia*

E. alpina var. *alpina*			*E. rubra*							Intermediate morphology		*E. alpina* var. *carmelitana*	
	**248**	**259**	**266**	**257**	**263**	**265**	**224**	**269**	**274**	**229**	**233**	**340**	**321**
**234**	0.01	0.07	0.03	0.16	0.10	0.13	0.30	0.12	0.15	0.01	0.05	0.16	0.21
**248**		0.05	0.02	0.21	0.14	0.20	0.35	0.18	0.20	0.03	0.06	0.21	0.23
**259**			0.03	0.25	0.18	0.23	0.39	0.22	0.25	0.11	0.13	0.20	0.24
**266**				0.23	0.17	0.20	0.39	0.19	0.23	0.06	0.10	0.20	0.22
**257**					0.08	0.07	0.28	0.07	0.07	0.12	0.09	0.23	0.27
**263**						0.11	0.23	0.06	0.02	0.05	0.06	0.20	0.23
**265**							0.25	0.02	0.08	0.10	0.01	0.17	0.23
**224**								0.20	0.24	0.25	0.27	0.36	0.34
**269**									0.06	0.07	0.08	0.16	0.21
**274**										0.09	0.06	0.24	0.27
**229**											0.03	0.18	0.20
**233**												0.17	0.22
**340**													0.06

**Figure 4.**
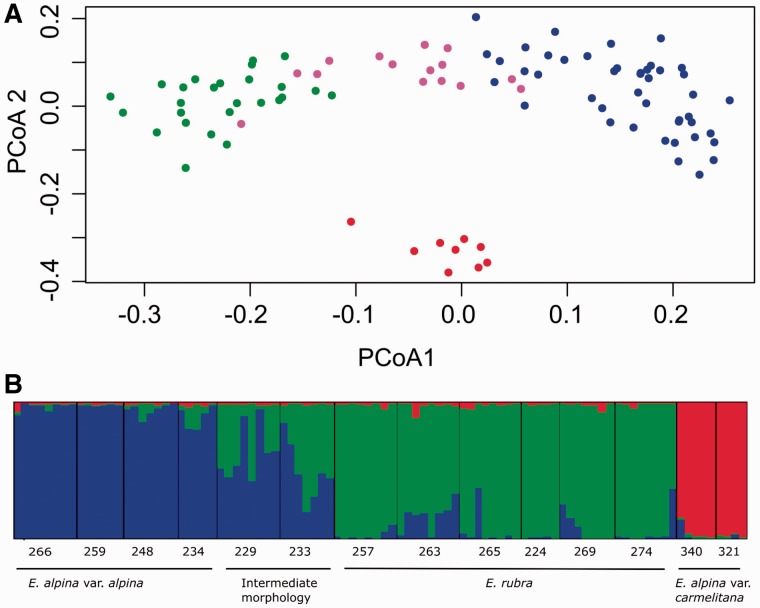
(A) PCoA of AFLP among 94 *Escallonia* individuals. The first 2 axes represented in the figure explain 18.65 and 10 % of total variability. Colours indicate groups according to taxonomy: red: *E. alpina* var. *carmelitana*; green: *E. rubra*; blue: *E. alpina* var. *alpina*; fuchsia: IM. (B) Genetic structure inferred from bayesian analysis using STRUCTURE software; bars represent the proportion of individuals assigned to each of three genetic clusters (*K* = 3).

The distribution of individuals among the first and second principal coordinates of the PCoA analysis is shown in Figure 4A; the first 2 eigenvalues are 0.19 and 0.10, and both account for 28.65 % of total variation. *Escallonia.* *rubra* and *E. alpina* individuals are separated along the first axis, while individuals that share morphological diagnostic characters of both species are spread in an intermediate position (Fig. 4). Along the second axis, *E.* *alpina* var. *carmelitana* populations are discretely grouped and well separated from the rest of *E. alpina* and *E. rubra* populations.

STRUCTURE results are shown in Figure 4B. Individuals were assigned to three genetic groups, as suggested by Evanno’s method, mostly corresponding with taxonomy: *E.* *rubra, E. alpina* var. *alpina* and *E.* *alpina* var. *carmelitana*. Some individuals from *E. alpina* var. *alpina* and *E. rubra* populations had a small degree of admixture (e.g. in populations 263 and 269). Populations of IM (229 and 233) showed a mixed genetic contribution from *E. alpina* var. *alpina* and *E. rubra*. On the contrary, individuals from *E. alpina* var. *carmelitana* (populations 321 and 340) showed almost no signal of genetic admixture.

AMOVA analysis (Table 3) revealed a significant level of differentiation between *E. rubra, E. alpina* var. *alpina* and *E. alpina* var. *carmelitana* (25.67 % *P* < 0.001). The highest percentage of variation was found within populations (63.65 %, *P* < 0.001).

**Table 3.** Results of the AMOVA for 12 populations of *alpina var.* *E.* *alpina, E. alpina var.* *carmelitana and E. rubra*, based on AFLP data. The analysis was performed to test differences among three groups. Degrees of freedom (d.f.), sum of squares deviations (SSD), variance components (VC), percentage of total variance (% total) and significance value (*P*) are given for each hierarchical level.

Source of variation	d.f.	SSD	VC	% total	*P*-value
Amongst groups	2	375.919	6.860	25.67	<0.001
Amongst populations within groups	9	324.886	2.851	10.67	<0.001
Within populations	67	1139.600	17.009	63.65	<0.001
Total	78	1840.405	26.721		

### Leaf shape

Morphological examination allowed us to characterize leaves of *E. alpina* var. *carmelitana* as narrowly obovate to spatulate, lanceolate, tapering gradually to the base, with the apex acute to obtuse, rarely truncate (see Fig. 2C), while those of the type variety are mostly obovate, sometimes suborbicular-cuneate or spatulate-lanceolate, with the apex typically obtuse, sometimes subacute, rarely truncate or invaginated (see Fig. 2B).

**Figure 5.**
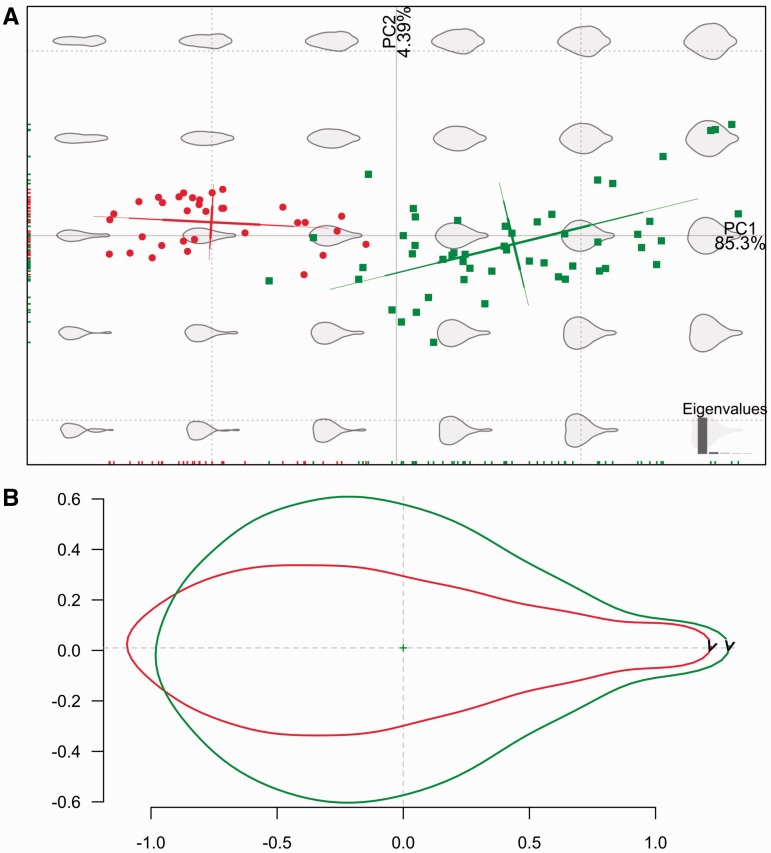
(A) Scatter plot of PCs one and two from geometric morphometrics analysis using Elliptic Fourier descriptors for leaves of *E. alpina* var. *alpina* (green) and *E. alpina* var. *carmelitana* (red). Figures in the background show reconstructions of leaf shape according to each position in the PCs space. (B) Mean shape of leaves assigned to *E. alpina* var. *E. alpina* (green) and *alpina* var. *carmelitana* (red) according to Elliptic Fourier analysis.

Geometric morphometry further reinforces these observations (Fig. 5A and B); the PCA of the Elliptic Fourier descriptors shows two groups of leaves that correspond to E. *alpina* var. *alpina* and *E. alpina* var. *carmelitana*, although they are partially overlapped (Fig. 5A). The first two PCs retained 89.7 % of total variation. As seen from the reconstruction of the shapes along the first axis, the major source of leaf outline variation is anisotropy (length to width ratio). The mean leaf shape for each group is shown in Figure 5B. Cross-validation performed over LDA values (using the first nine PCs) was highly successful for both varieties, as 93 % of all leaves was well classified (*E. alpina* var. *alpina* 94.4 % and *E. alpina* var. *carmelitana* 91.7 %). A MANOVA test also showed significant differences between the two varieties (*P* < 0.001).

## Discussion

### Southern populations show genetic admixture and could be the result of hybridization between *E. alpina* var. *alpina* and *E. rubra*

The results of our analyses at the population level support preliminary evidence of genetic admixture (Morello *et al.* 2013) and the morphological hypothesis of Sleumer (1968) of natural hybridization between *E.* *alpina* and *E. rubra*. Two populations (229 and 233, Table 1, Figs. 1 and 2D) from the southern limit of the distribution range of both species (southwestern Santa Cruz province, Argentina, *ca.* 50° S), exhibited intermediate genetic characters and low genetic distance from *E. rubra* and *E. alpina* var. *alpina*; accordingly, both collections showed IM between *E. alpina* (solitary flowers, Fig. 2B) and *E. rubra* (stipitate glands on hypanthium, pedicels and young stems, and resinous spots on the adaxial leaf surface, Fig. 2A). We were able to identify herbarium collections with IM from the same geographic location **[Supporting Information 1a]**; moreover, most of the collections identified by Sleumer (1968) as natural hybrids are located in southwestern Santa Cruz in the proximity of Lago Argentino. Accidental interspecific cross pollination is likely to occur where more than one species are in sympatry and share pollinators. Floral biology could help elucidate the origin of the hybrid populations. To date, there are few studies on the floral biology of *Escallonia* species; Valdivia and Niemeyer (2006) found that *E. myrtoidea* is commonly visited by generalist pollinators such as bees and this is in accordance with our observations in the field. Although the parental species have also been recorded in the same region (Sleumer 1968), we only observed and collected populations with IM.

New questions arise if we assume these southern populations are indeed of hybrid origin. Why are hybrids more common in the southernmost locations? And why are these populations mostly composed by hybrids? One plausible answer is that hybrid fitness could be dependent on habitat: in most of the parental distribution range, hybrid fitness may be significantly lower than their parental fitness, promoting isolation. On the other hand, in marginal habitats selective pressures are different, and hybrids could be just as fit or even favoured by natural selection. In our study, IM populations were found in the southern limit of both parental species distribution range. The role for hybridization in evolution in marginal or altered habitats has been discussed in previous works Burke and Arnold (2001), and Rieseberg *et al.* (2003). A similar process could have occurred if parental species had shared glacial refugia. Reproductive isolation mechanisms that may have arisen during and after speciation could have been lost later as a consequence of reduced or fragmented habitats and climatic changes. This scenario has been proposed for plant species in Europe (e.g. Heuertz *et al.* 2006; Palmé *et al.* 2004) and as an explanation for hybrid *Nothofagus* trees in southern South America (Acosta and Premoli 2010; Soliani *et al.* 2012). Several studies, either in plants or animals, support high biological diversity in southern Patagonia. Phylogeographic studies showed populations with high diversity at high latitudes (ca. 50° S), likely being the result of *in situ* survival during Pleistocene glaciations (e.g. *Hordeum*: Jakob *et al.* 2009; *H.* *incana*: Tremetsberger *et al.* 2009; *C.* *polyrhiza*: Cosacov *et al.* 2010; *Nothofagus pumilio*: Mathiasen and Premoli 2010; *Podocarpus nubigena*: Quiroga and Premoli 2010; *Mulinum spinosum*: Sede *et al.* 2012; many of them compiled and analyzed in Sérsic *et al.* 2011). Furthermore, Domínguez *et al.* (2006) described an area of high endemism in southwestern Santa Cruz province.

The fact that current hybrid populations are composed mostly by admixtured individuals suggests that gene flow was not an isolated event in the region and it may have played a significant role during the evolution of *Escallonia* species; this could be relevant for reconstructing processes as demographic contractions and expansions, colonization routes and possible survival in refugia. A more comprehensive phylogeographycal analysis is necessary to answer questions on the history and origin of these putative hybrid populations, and an experimental design would be useful to test hypotheses concerning hybridization mechanisms.

### *E.* *alpina* var. *alpina* and *E. alpina* var. *E.* *carmelitana* are separate lineages

*E.* *alpina* var. *alpina* and *E. alpina* var. *carmelitana* were not grouped together: the type variety was more closely related to *E. rubra* than it was to var. *carmelitana*. This finding was supported by DNA sequence variation and AFLP markers.

According to the protologue (Kausel 1953), *E.* *alpina* var. *carmelitana* was differentiated from the type variety by the leaf size (which is highly variable even within individuals) and stem colour (which is affected by age and state of preservation). During the morphological study of collections of *E.* *alpina* var. *carmelitana* we observed differences in leaf shape rather than size. Leaf shape differences were strongly corroborated by geometric morphometrics analysis and this new character will be useful for species identification purposes.

*E.* *alpina* var. *carmelitana* is a clearly distinct group, with genetic and morphological differences and with a restricted geographic distribution, which deserves to be considered an independent entity. Populations of *E. alpina* var. *carmelitana* are located between 35° and 38° S, 70° W: this region is characterized by two high mountain ranges (the Andes and Cordillera del Viento), volcanoes, e.g. Copahue, and valleys; the zone is well irrigated, with several tributaries of the Neuquén river, although there are only small lakes, e.g. Varvarco Campos and Caviahue (Bran *et al.* 2002). A high level of endemism has historically been reported for this zone of northern Patagonia (Cabrera and Willink 1973; Simpson and Neff 1985). Moreover, it has been proposed that high genetic diversity of populations in northern Patagonia may be a consequence of *in situ* survival in refugia during glacial cycles (*Austrocedrus.* *chilensis*: Arana *et al.* 2010; *Podocarpus.* *nubigena*: Quiroga and Premoli 2010; *Anarthrophyllum desideratum*: Cosacov *et al.* 2013, and more examples in Sérsic *et al.* 2011).

Nevertheless, the strong pattern of genetic, morphological and geographical differentiation found in *E. alpina* var. *alpina* vs *E. alpina* var. *carmelitana* do not seem to be a consequence of recent periods of isolation and expansion due to climatic events during the Quaternary. High mountains may have acted as dispersal barriers (Struwe *et al.* 2009) promoting isolation and vicariance, even generating deep divergence among species and high levels of biological diversity (Hughes and Eastwood 2006; Pennington *et al.* 2010). Sérsic *et al.* (2011 and literature herein) found shared patterns of genetic distribution along the Andes, both for plants and animals: particularly, a high diversity zone around 35°–39° S. It has been hypothesized that many Patagonian plants have diversified in this region as a consequence of Miocene-Pliocene orogeny and associated tectonic processes; e.g. *Calceolaria.* *polyrhiza* (Cosacov *et al.* 2010), *Hordeum* (Jakob *et al.* 2009) and *Hypochaeris. incana* (Tremetsberger *et al.* 2009). Our results, combining morphology, plastid sequences and AFLP, together with the geographically structured phylogenies of *Escallonia* support the hypothesis that Andean orogeny has played an important role in the diversification of the genus.

## Conclusions

New evidence of genetic admixture in *Escallonia* populations might be a result of interspecific hybridization. Further studies on ecology, pollination and floral biology could help to understand the role of interspecific gene flow in the evolution of the genus. Moreover, a comprehensive phylogeographic work, using co-dominant markers, will give hindsight in the recent evolution of *Escallonia* species and their interaction over time.

Additionally, we conclude that *E. alpina* var. *alpina* and *E. alpina* var. *carmelitana* are distinct lineages, and taxonomy should be revised to reflect their separation. *Escallonia* species, as currently circumscribed, could be concealing a richer diversity. We expect that new studies, combining morphology and genetics, will improve our understanding of biological diversity in the genus and general trends of plant evolution in Patagonia.

## Accesion Numbers

All new sequences were deposited in GenBank with accession numbers KU759574- KU759579.

## Sources of Funding

DNA sequencing and fieldwork was aided by PIP 1122012010036CO National Scientific and Technical Research Council of Argentina (CONICET) granted to M. Morando.

## Contributions by the Authors

S.S. and S.M. conceived the idea; S.S. collected the samples; S.S. and S.M. performed the molecular analysis; S.M. performed the geometric morphometric analysis; S.S. and S.M. analysed the data and led the writing.

## Conflict of Interest Statement

None declared.

## Supplementary Material

Supplementary Data

## Supporting Information

The following additional information is available in the online version of this article —

**Supporting information: 1**. (a) Representative specimens of intermediate morphology between *E. rubra* and *E. alpina* from herbarium material, and (b) additional herbarium collections of *E. alpina* analyzed in geometric morphometrics analyses. **2**. Primers used in AFLP protocol and plastid DNA amplification and sequencing. **3**. AFLP data matrix. **4**. Digitized outlines of *E. alpina* leaves used for geometric morphometrics analyses.
