# Supplementary material for: Genetic admixture and lineage separation in a southern Andean plant
Source: AoB Plants. 2016 Jul 11;8:plw034. doi: 10.1093/aobpla/plw034 (PMC4940511; doi:10.1093/aobpla/plw034)
Supplement: Supplementary Data [file supp_8_plw034_index.html]

Genetic admixture and lineage separation in a southern Andean plant — Supplementary Data 

# Genetic admixture and lineage separation in a southern Andean plant

## Supplementary Data

files

- Supplementary Data - docx file
- Supplementary Data - txt file
