# Supplementary material for: Genetic admixture and lineage separation in a southern Andean plant
Source: AoB Plants. 2016 Jul 11;8:plw034. doi: 10.1093/aobpla/plw034 (PMC4940511; doi:10.1093/aobpla/plw034)
Supplement: Supplementary Data [file supp_plw034_aobplants-15348-s03.docx]

**Supporting Information**

**1**. Representative specimens of intermediate morphology between *E. rubra* and *E. alpina* from herbarium material (a) and additional herbarium collections of *E. alpina* analysed in geometric morphometric analyses (b).

1. Argentina. **Santa Cruz**: Dept. Lago Argentino, Parque Nacional Los Glaciares, Camping Arroyo Correntoso, 50° 29’ 14.6’’ S, 72° 57’ 30’’ W, 263 m, 30 Jan 2003, *Coccuci & Sérsic 2496* (CORD, SI); Parque Nacional Los Glaciares, 3 Feb 1955, *Pérez Moreau 69850* (BA, SI); Lago Argentino, cerca del glaciar Spegazzini, bosque al borde del Lago, 19 Jan 1967, *Boelcke et al. 12608, 12609, 12613* (BAA, BAB, SI); Lago Argentino, brazo norte, ventisquero Upsala, Bahía Ciprés, 50° 05’ S, 73° 17’ W, 19 Jan 1967 (BAA, BAB, SI).
2. Argentina. **Mendoza**: Malargüe, Río Grande, a 10 km de Azufrera, 22 Jan 1982, *Cabrera et al. 33501* (SI); Uspallata, 2,200 m, 25 Feb 1903, *Buchtien s.n.* (P, SI). **Neuquén**: Loncopué, arroyo Chenque Pehuén, 25 km W de Loncopué, 1,800m, 15 Feb 1974, *Gentile 200* (SI); Chenque Pehuén, 16 Jan 1965, *Schajovakoy I36/IV* (SI); Ñorquín, Río Ñireco, 18 km W RN 40, 39º 08’ S, 70º 31’ W, 1,800 m, 24 Jan 2002, *Ezcurra et al. 3278* (BURU); Minas, 11 km al W de Las Ovejas por RP 45 en dirección a Lagunas de Epulauquen, Río Nahueve, 36° 58’ 42’’ S, 70° 52’ 23’’ W, 1,311 m, 31 Dec 2009, *Sede & Calcagno* *215* *a-c* (SI).

Chile. **X Región de Los Lagos**: Osorno, Parque Nacional Puyehue, Volcán Casablanca, cráter Raihuén, 1,264 m, 40° 46’ 44’’ S, 72° 11’ 32’’ W, 18 Feb 2010, *Sede & Calcagno 284* *a-h* (SI).

**2**. Primers used in AFLP protocol and plastid DNA amplification and sequencing.

AFLP- Adaptors

EcoRI 5’-CTCGTAGACTGCGTACC-3’

3’-CTGACGCATGGTTAA-5’

MseI 5'-GACGATGAGTCCTTGAG-3'

3'-TACTCAGGAACTCAT-5'

AFLP- Preselective amplification

EcoRI+1 5'-GACTGCGTACCAATTCA-3'

MseI+1 5’-GATGAGTCCTTGAGTAAC-3'

AFLP- Selective amplification

EcoRI+3 5'-GACTGCGTACCAATTCACT-3’

MseI+3 5'-GATGAGTCCTTGAGTAACAC-3

*trnS-trnG* intergenic spacer

trnS^GCU^ AGATAGGGATTCGAACCCTCGGT

5´trnG2S TTTTACCACTAAACTATACCCGC

(Shaw *et al.* 2005)

3′*trnV-ndhC* intergenic spacer

trnV^(UAC)^×2 GTCTACGGTTCGARTCCGTA

ndhC TATTATTAGAAATGYCCARAAAATATCATATTC

(Shaw *et al.* 2007)

*NdhF* gene

5F ATGGAACAGACATATCAATATGCGTGG

972R CATCATATAACCCAATTGAGAC

(Olmstead and Sweere 1994)

1230F ACCCCTTGCTTGTTTTTGG

2357R CTCTTGTGACCCTTCTTTTCG

(Sede *et al.* 2013)
